# Novel positive allosteric modulators of GABA_A_ receptors with anesthetic activity

**DOI:** 10.1038/srep25943

**Published:** 2016-05-20

**Authors:** Maria C. Maldifassi, Roland Baur, David Pierce, Anahita Nourmahnad, Stuart A. Forman, Erwin Sigel

**Affiliations:** 1 Institute of Biochemistry and Molecular Medicine, University of Bern, CH-3012 Bern, Switzerland; 2 Department of Anesthesia, Critical Care and Pain Medicine, Massachusetts General Hospital, Boston, 02114 Massachusetts

## Abstract

GABA_A_ receptors are the main inhibitory neurotransmitter receptors in the brain and are targets for numerous clinically important drugs such as benzodiazepines, anxiolytics and anesthetics. We previously identified novel ligands of the classical benzodiazepine binding pocket in α_1_β_2_γ_2_ GABA_A_ receptors using an experiment-guided virtual screening (EGVS) method. This screen also identified novel ligands for intramembrane low affinity diazepam site(s). In the current study we have further characterized compounds 31 and 132 identified with EGVS as well as 4-O-methylhonokiol. We investigated the site of action of these compounds in α_1_β_2_γ_2_ GABA_A_ receptors expressed in *Xenopus laevis* oocytes using voltage-clamp electrophysiology combined with a benzodiazepine site antagonist and transmembrane domain mutations. All three compounds act mainly through the two β+/α**−** subunit transmembrane interfaces of the GABA_A_ receptors. We then used concatenated receptors to dissect the involvement of individual β+/α**−** interfaces. We further demonstrated that these compounds have anesthetic activity in a small aquatic animal model, *Xenopus laevis* tadpoles. The newly identified compounds may serve as scaffolds for the development of novel anesthetics.

The search for novel anesthetics has been triggered by the rising age of patients and increasing use of anesthesia outside the operating room^1^,^2^. A key site of action of the potent anesthetics propofol and etomidate is the major inhibitory receptor in the mammalian central nervous system, the γ-aminobutyric acid type A (GABA_A_) receptor. These receptors are composed of five homologous subunits organized around a central Cl^−^ selective channel^3^. Each subunit contains a large N-terminal extracellular domain (ECD), a transmembrane domain (TMD) with four alpha-helices (TM1 to TM4), and a variable-length intracellular domain (ICD) between TM3 and TM4. 19 subunits of the GABA_A_ receptor have been cloned (for review see: 4, 5, 6), denoting that numerous types of receptor isoforms exist^5^. The most abundant GABA_A_ receptor in the brain comprises α_1_, β_2_ and γ_2_ subunits^3^,^4^,^5^,^7^. The receptor possesses a 2α:2β:1γ subunit stoichiometry^8^,^9^,^10^,^11^, with a subunit arrangement of γβαβα anti-clockwise as seen from the synaptic cleft^10^,^11^,^12^,^13^. The receptor composition and arrangement influence its pharmacological properties^14^,^15^.

Benzodiazepines modulate α_1_β_2_γ_2_ GABA_A_ receptor function by binding to a high affinity site located at the α+/γ− ECD interface, homologous to the agonist binding sites at β+/α− ECD interfaces^16^,^17^. In addition to the high affinity binding site for benzodiazepines (site 1), there are other low affinity sites. One of these, site 2, is located at the ECD α+/β− interface^18^,^19^. Others, together designated as site 3, are located in the TMD, based on abolition of benzodiazepine effects by combined isoleucine substitutions at the homologous residues α_1_S269, β_2_N265, and γ_2_S280^20^.

GABA_A_ receptors are also targets for potent intravenous anesthetics, including barbiturates, propofol and etomidate^21^,^22^,^23^,^24^,^25^. Interestingly, receptor sensitivity to intravenous anesthetics is affected by benzodiazepine site 3 mutations^26^,^27^,^28^,^29^,^30^. Diverse anesthetics not only potentiate GABA-induced Cl^−^ currents, but additionally at high concentrations directly activate GABA_A_ receptors^31^,^32^. Photo-affinity labeling has located allosteric sites for the intravenous anesthetics etomidate and propofol to the TM1 of α and TM2, TM3 of β subunits^23^,^24^,^33^.

Previously, we reported a new method to identify ligands of the high affinity benzodiazepine pocket, experimental-guided virtual screening (EGVS), integrating experimental data with homology modeling of the GABA_A_ receptor^34^. EGVS identified some ligands that only recognized site 1, others that recognized both site 1 and site 3^35^, and another set that only recognize site 3.

Here we describe the actions of two compouds identified by EGVS, 31 and 132^34^ and 4-O-methylhonokiol^36^. Using mutations and concatenated receptors we determined that the three compounds act mainly through the TMD β+/α− interfaces (site 3), and particularly the γβ+/α−β site. The anesthetic action of these drugs was explored *in vivo*, revealing potencies similar to propofol.

## Results

In an attempt to find novel ligands for the high affinity site for benzodiazepines on GABA_A_ receptors we screened 198 compounds for displacement of the high affinity benzodiazepine site (called site 1 previously) antagonist [^3^H]-Ro 15-1788 at receptors expressed in HEK-cells. Many high affinity ligands were identified^34^. One compound, SJM3 acted as antagonist with high affinity at site 1, but allosterically potentiated receptor activation through sites in the membrane (called sites 3 previously)^35^. Compounds 31 and 132 either did not or weakly displaced [^3^H]-Ro 15-1788 from site 1 but potently enhanced GABA_A_ receptor activation. 4-O-methylhonokiol shares these characteristics, potentiating α_1_β_2_γ_2_ receptors with an EC_50_ of 5.4 ± 1.8 μM, independent of the high affinity site for benzodiazepines^36^. Here, we report mechanistic and animal studies of these three compounds.

### Compounds 31 and 132 are allosteric modulators of α_1_β_2_γ_2_ GABA_A_ receptors

**Figure 1:**
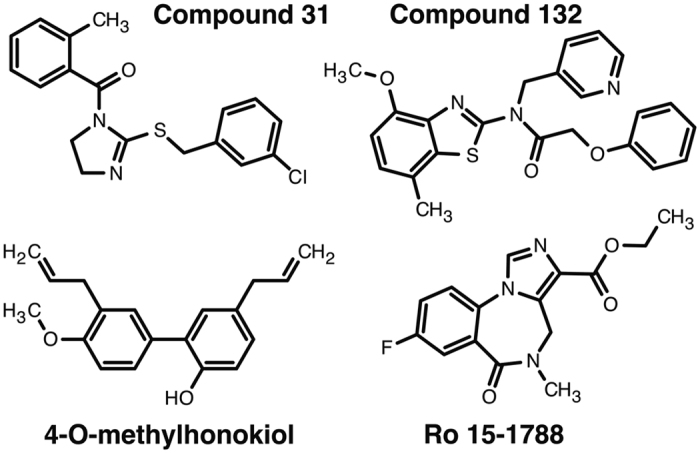
Chemical structure of compounds 31, 132, 4-O-methylhonokiol, and the high-affinity benzodiazepine antagonist Ro 15-1788.

First, we investigated if compounds 31 and 132 were able to act as agonists. Figure 1 shows their chemical structures compounds. Both compounds at 3 and 30 μM elicited only very small currents by themselves in α_1_β_2_γ_2_ GABA_A_ receptors expressed in *Xenopus* oocytes. These compounds elicited at the concentration of 3 μM and 30 μM currents amounting to 0.1 ± 0.06% (mean ± SD, n = 3) and 0.3 ± 0.13% (mean ± SD, n = 3), respectively of the maximal current amplitude elicited by GABA in the same oocytes. Thus neither of the compounds tested acts as an appreciable agonist on α_1_β_2_γ_2_ receptors.

**Figure 2:**
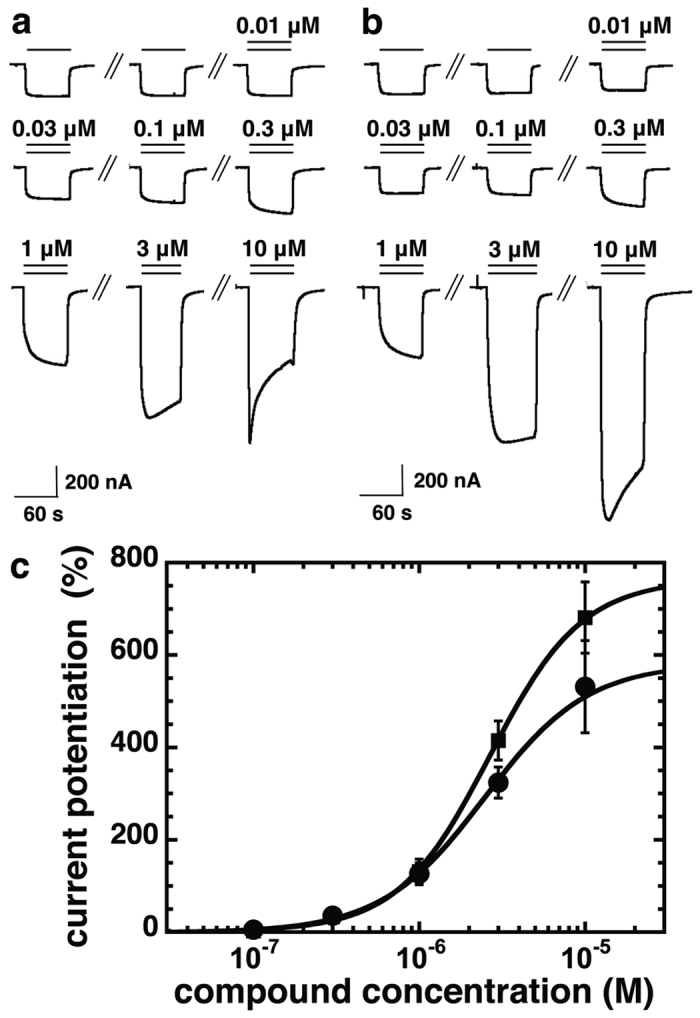
Compounds 31 and 132 stimulate GABA currents mediated by α1β_2_γ_2_ receptors in a concentration-dependent manner. α_1_β_2_γ_2_ receptors were expressed in *Xenopus* oocytes and electrophysiological experiments were performed. Original current traces of an experiment with compound 31 (**a**) and with compound 132 (**b**). Numbers indicate applied concentrations of the respective compounds. (**c**) Concentration dependence of the positive allosteric modulation by compound 31 (closed circles) and compound 132 (closed squares) in oocytes expressing α_1_β_2_γ_2_ receptors. Mean data ± SD for both compounds is shown, n = 3.

Both compounds strongly enhanced currents elicited by GABA. We established the concentration response curves of this positive allosteric modulation. After two applications of GABA at a concentration eliciting 0.5–1.5% of the maximal current amplitude, the same concentration of GABA was co-applied with increasing concentrations of the tested compounds. Figure 2a,b show current traces demonstrating positive modulation by different concentrations of compounds 31 and 132, respectively. Figure 2c summarizes the results of three of such experiments. Both compounds potentiate GABA elicited currents in oocytes expressing α_1_β_2_γ_2_ receptors. For compound 31, at high concentrations apparent desensitization was observed, that could be partly due to open channel blocker effect. For both compounds no saturation at the highest concentration was obtained, because of poor solubility we could not test higher concentrations.

### Potentiation by compounds 31 and 132 is not affected by Ro 15-1788

**Figure 3:**
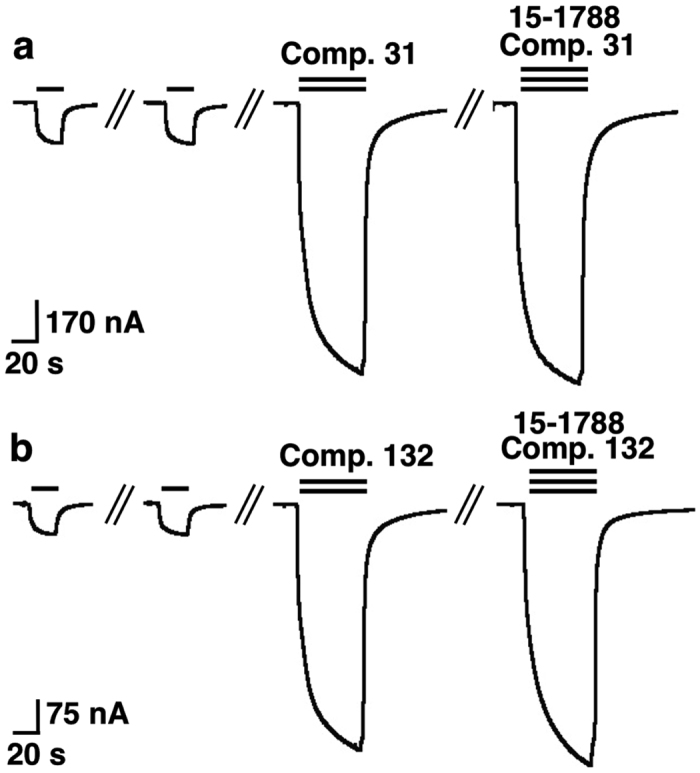
Compounds 31 and 132 do not act at the classical high affinity site for benzodiazepines. GABA at a concentration eliciting 0.5% of the maximal current amplitude (EC_0.5_, single bars) was applied until a stable response was obtained. Subsequently, the same concentration of GABA was co-applied with 3 μM of compounds 31 (**a**) or 132 (**b**), which resulted both in a large increase of current amplitude. Co-application of Ro 15-1788 with compound and GABA did not reduce the degree of modulation in both cases. Experiments were repeated 4 times, with three different batches of oocytes, with a similar outcome.

From the binding data we did not expect that the two compounds are acting through the high affinity benzodiazepine binding site 1 in α_1_β_2_γ_2_ receptors. Nevertheless, we tested whether 1 μM of the site 1 antagonist Ro 15-1788 inhibits potentiation of GABA currents by compounds 31 or 132. Either compound (3 μM) strongly potentiated currents elicited by GABA. Potentiation by either drug was not inhibited by 1 μM Ro 15-1788 (Fig. 3). Relative current amplitudes in the presence vs. absence of the antagonist were 112 ± 8% (mean ± SD, n = 4, p > 0.05, *t test*) for compound 31, and 115 ± 14% (mean ± SD, n = 4, p > 0.05, *t test*) for compound 132. This confirms that potentiation by compounds 31 and 132 does not result from action at the benzodiazepine site 1.

### Compounds 31 and 132 and 4-O-methylhonokiol act at the low affinity benzodiazepine site 3 in α_1_β_2_γ_2_ receptors

**Figure 4:**
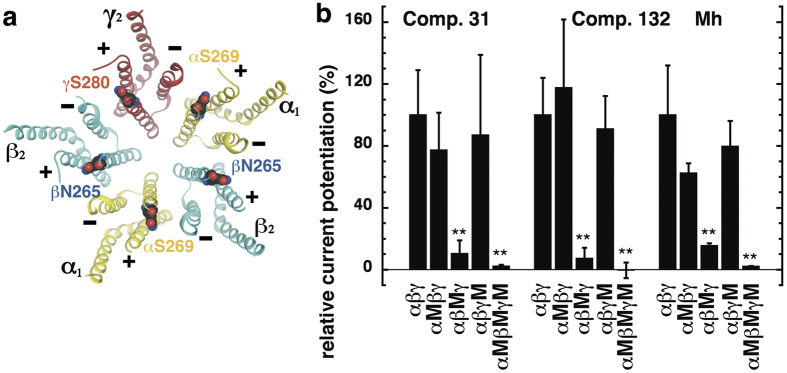
(**a**) Model structure of the GABA_A_ receptor transmembrane domain. The major isoform of the GABA_A_ receptor is composed of two α_1_, two β_2_, and one γ_2_ subunits. The model structure depicts the crystalized homomeric β_3_ GABA_A_ receptor (PDB structure 4COF)^38^. In this figure, some of the β_3_ subunits were renamed α_1_ (yellow), β_2_ (blue) and γ_2_ (red); structures are shown in ribbon representation. The mutated residues α_1_S269, β_2_N265, and γ_2_S280 are located at the interfaces between subunits. (**b**) Potentiation of the GABA response by compound 31 (3 μM), compound 132 (3 μM), and 4-O-methylhonokiol (1 μM, abbreviated Mh) in wild-type α_1_β_2_γ_2_, single mutant (α_1_M, β_2_M, γ_2_M), and triple mutant receptors expressed in *Xenopus* oocytes. The bars indicate mean ± SD, n = 3.

We next investigated whether benzodiazepine site 3 TMD mutations affect potentiation by compounds 31 and 132. Combining three homologous site 3 mutations in α_1_β_2_γ_2_ receptors, α_1_S269I, β_2_N265I and γ_2_S280I, eliminated the potentiation by high concentrations of diazepam^20^. We investigated the effects of these mutations individually and combined, abbreviating them as α_1_M, β_2_M and γ_2_M. Recently, we described the potency of GABA to activate all of these receptor subtypes expressed in *Xenopus* oocytes^37^. In order to exclude general gating effects caused by these mutations we showed that potentiation by low concentrations of diazepam and by THDOC are not affected^37^. For illustration, the localization of the mutations is shown in the crystalized homomeric β_3_ receptor^38^ where some of the β_3_ subunits were renamed α_1_, β_2_ and γ_2_ (Fig. 4a).

Wild-type α_1_β_2_γ_2_, α_1_Mβ_2_γ_2_, α_1_β_2_Mγ_2_, α_1_β_2_γ_2_M and α_1_Mβ_2_Mγ_2_M receptors were expressed in *Xenopus* oocytes. Using electrophysiological techniques we determined the effect of these point mutations on the potentiation by compounds 31, 132 and 4-O-methylhonokiol, normalizing to the potentiation in wild type α_1_β_2_γ_2_ receptors.

Figure 4b summarizes the results obtained. The single mutations in the α_1_ and in the γ_2_ subunits did not significantly alter the degree of potentiation by 3 μM of either compound 31 or 132. Relative to wild-type α_1_β_2_γ_2_ receptors, modulation in mutated α_1_Mβ_2_γ_2_ receptors by compound 31 amounted to 77 ± 24% (mean ± SD, n = 3, p > 0.05, *Tukey posthoc test*), and by compound 132 to 118 ± 44% (mean ± SD, n = 3, p > 0.05, *Tukey posthoc test*). Modulation in α_1_β_2_γ_2_M receptors by compound 31 was 87 ± 52% (mean ± SD, n = 3, p > 0.05, *Tukey posthoc test*), and by compound 132 was 91 ± 21% (mean ± SD, n = 3, p > 0.05, *Tukey posthoc test*). In contrast, modulation by both compounds was strongly impaired in α_1_β_2_Mγ_2_ and triply mutated α_1_Mβ_2_Mγ_2_M receptors. Potentiation by 3 μM compound 31 in α_1_β_2_Mγ_2_ was 10.4 ± 8.5% (mean ± SD, n = 3, p = 0.007, *Tukey posthoc test*), and in α_1_Mβ_2_Mγ_2_M receptors was 2.3 ± 0.8% (mean ± SD, n = 3, p = 0.0043, *Tukey posthoc test*). Potentiation by compound 132 relative to that in α_1_β_2_γ_2_ was also dramatically reduced in α_1_β_2_Mγ_2_ 7.3 ± 6.8% (mean ± SD, n = 3, p = 0.003, *Tukey posthoc test*) and α_1_Mβ_2_Mγ_2_M −0.4 ± 5.1% (mean ± SD, n = 3, p = 0.0021, *Tukey posthoc test*), respectively.

Potentiation by 1 μM 4-O-methylhonokiol was also significantly reduced only in α_1_β_2_Mγ_2_ and α_1_Mβ_2_Mγ_2_M receptors, similar to compounds 31 and 132. Relative to wild-type α_1_β_2_γ_2_ receptors, modulation in α_1_Mβ_2_γ_2_ mutated amounted to 63 ± 6% (mean ± SD, n = 3, p > 0.05, *Tukey posthoc test*), and in α_1_β_2_γ_2_M receptors to 79 ± 16% (mean ± SD, n = 3, p > 0.05, *Tukey posthoc test*). Relative to α_1_β_2_γ_2_, residual potentiation in α_1_β_2_Mγ_2_ receptors amounted to 15.6 ± 1.5% (mean ± SD, n = 3, p = 0.0022, *Tukey posthoc test*), and to 2.2 ± 0.2% (mean ± SD, n = 3, p = 0.0018, *Tukey posthoc test*) in α_1_Mβ_2_Mγ_2_M.

The above data suggests that the modulatory site(s) for the three compounds we studied is located in one or both of the β+/α− TMD subunit interfaces on α_1_β_2_γ_2_ GABA_A_ receptors.

### Role of the individual β+/α− subunit interfaces in channel modulation by compounds 31, 132 and 4-O-methylhonokiol

**Figure 5:**
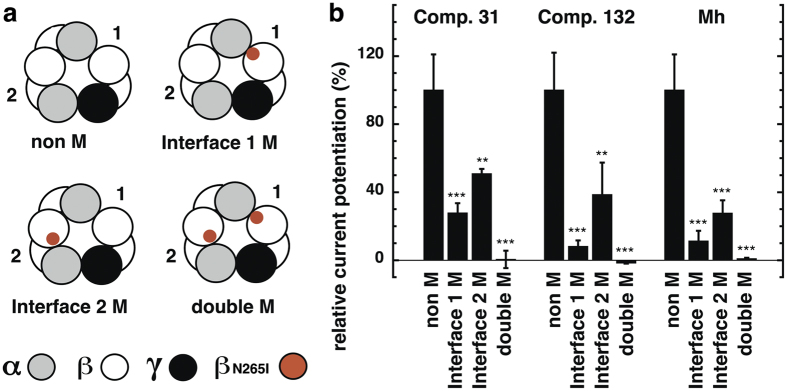
Individual roles of the two β+/α− interfaces in channel modulation by compounds 31, 132 and 4-O-methylhonokiol. (**a**) Scheme showing the four concatenated wild-type and mutant receptors. 1 and 2 refer to the two different β+/α− subunit interfaces, interface 1 and interface 2. The location of the β_2_N265I mutations is indicated in red color. Concatenated receptors were prepared containing no mutation (α_1_-β_2_-α_1_/γ_2_-β_2_, non M), a mutation at interface 1 (α_1_-β_2_-α_1_/γ_2_-β_2_M, interface 1 M), a mutation at interface 2 (α_1_-β_2_M-α_1_/γ_2_-β_2_, interface 2 M), or mutations in both sites (α_1_-β_2_M-α_1_/γ_2_-β_2_M, double M). Interface 2 harbors a binding site for GABA with higher apparent affinity for channel gating than the one positioned at the interface 1^54^. (**b**) Potentiation by compound 31 (3 μM), compound 132 (3 μM), and 4-O-methylhonokiol (1 μM), using an EC_0.5–1.5_ concentration of GABA for each concatenated receptor subtype. Bars indicate mean ± SD, n = 3.

Each GABA_A_ receptor contains two β+/α− subunit interfaces. Combined mutation at these interfaces greatly reduces the modulatory effects of compounds 31, 132 and 4-O-methylhonokiol. Using α_1_-β_2_-α_1_ and γ_2_-β_2_ subunit concatemers, we studied the effects of individual mutated interfaces. We designated receptors containing the mutant in the γ_2_-β_2_ construct interface 1 M, and the mutation in the α_1_-β_2_-α_1_ construct interface 2 M (Fig. 5a). The α_1_-β_2_M-α_1_ and γ_2_-β_2_M constructs were built, and were co-expressed with non-mutated dual or triple subunit constructs forming α_1_-β_2_-α_1_/γ_2_-β_2_M and α_1_-β_2_M-α_1_/γ_2_-β_2_ receptors. Both constructs were expressed together to form the double mutant receptor α_1_-β_2_M-α_1_/γ_2_-β_2_M.

Wild type concatenated receptors α_1_-β_2_-α_1_/γ_2_-β_2_ were also expressed. This receptor has an EC_50_ for GABA of approximately 120 μM^12^. Results are standardized to the potentiation observed in α_1_-β_2_-α_1_/γ_2_-β_2_ concatenated receptors. As shown in Fig. 5b, in the double mutant concatemeric receptors (α_1_-β_2_M-α_1_/γ_2_-β_2_M) potentiation for all three compounds was abolished. Relative to α_1_-β_2_-α_1_/γ_2_-β_2_ concatenated receptors, in the double mutant α_1_-β_2_M-α_1_/γ_2_-β_2_M receptor modulation by compound 31 was reduced to 1 ± 5% (mean ± SD, n = 3, p < 0.0005, *Tukey posthoc test*), by compound 132 to −2 ± 0.4% (mean ± SD, n = 3, p = 0.0001, *Tukey posthoc test*), and by 4-O-methylhonokilol to 1 ± 0.6% (mean ± SD, n = 3, p = 0.0001, *Tukey posthoc test*). In receptors containing only one mutation, either interface 1 or interface 2, modulation by compound 31 was reduced significantly compared to concatenated wild type receptors α_1_-β_2_-α_1_/γ_2_-β_2_. With interface 1 M, residual relative potentiation was 28 ± 6% (mean ± SD, n = 3, p = 0.0005, *Tukey posthoc test*) and with interface 2 M it was 51 ± 3% (mean ± SD, n = 3, p = 0.0059, *Tukey posthoc test*) of wild-type. Potentiation of interface 1 M and interface 2 M receptors differed significantly (p = 0.0035, *t test*). Therefore, both TMD β+/α− sites seem to contribute differently to modulation by compound 31, interface 1 being more efficacious.

Likewise, modulation by compound 132 was sensitive to the mutations at both sites. In interface 1 M receptors relative potentiation was reduced to 8 ± 4% (mean ± SD, n = 3, p = 0.0002, *Tukey posthoc test*), and in interface 2 M receptors to 39 ± 19% (mean ± SD, n = 3, p = 0.0035, *Tukey posthoc test*). Again, interface 1 M produced a larger impact than 2 M, although the difference was at the statistical limit (p = 0.0510, *t test*). Modulation by 4-O-methylhonokiol in interface 1 M and 2 M receptors was reduced to 11 ± 6% (mean ± SD, n = 3, p < 0.0001, *Tukey posthoc test*) and 28 ± 8% (mean ± SD, n = 3, p = 0.0004, *Tukey posthoc test*), respectively. The two mutations produced significantly different effects (p = 0.043, *t test*), with the interface 1 M effect again larger.

Therefore, potentiation by all three compounds displayed similar sensitivity patterns with homologous mutations in distinct β+/α− TMD sites of α_1_-β_2_-α_1_/γ_2_-β_2_ receptors. Both sites are necessary for full modulation, while interface 1 produces a larger impact than interface 2.

### Effect of the β_2_N265S mutation

**Figure 6:**
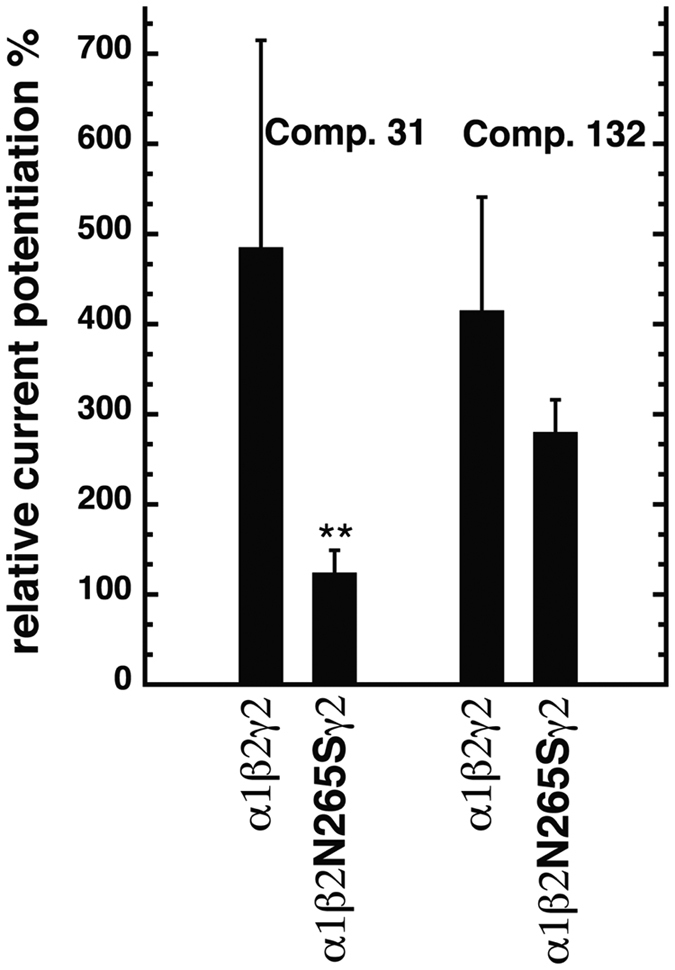
Effect of the β_2_N265S mutation on modulation by compounds 31 and 132. Wild-type α_1_β_2_γ_2_ and mutated α_1_β_2_N265Sγ_2_ receptors were expressed in *Xenopus* oocytes and studied. Potentiation of GABA currents was determined using 3 μM of compound 31 or 132. Bars indicate mean ± SD, n = 4–11.

The β_2_N265 residue is important for allosteric modulation of GABA_A_ receptors by many compounds acting through the TMD. This residue was initially described as a determinant for the modulatory action of loreclezole, where the β_2_N265S mutation created a receptor unresponsive to this compound^39^. Mutations in this residue also abolish potentiation by the anesthetics etomidate and propofol^28^,^40^. As shown in Fig. 6, the β_2_N265S mutation in α_1_β_2_γ_2_ receptors significantly reduced potentiation by 3 μM compound 31, from 485 ± 230% in wild-type receptors (mean ± SD, n = 11), to 125 ± 25% in the mutated receptor (mean ± SD, n = 4, p = 0.009, *Tukey posthoc test*). In contrast, this mutation did not significantly reduce potentiation by 3 μM compound 132: 415 ± 126% in wild-type receptors (mean ± SD n = 6) versus 280 ± 36% in the mutated receptor (mean ± SD, n = 4, p > 0.05, *Tukey posthoc test*). We have shown earlier that potentiation by 4-O-methylhonokiol in receptors carrying the β_2_N265S mutation was greatly reduced to about 40%^36^.

### Subunit specificity of compounds 31 and 132

**Figure 7:**
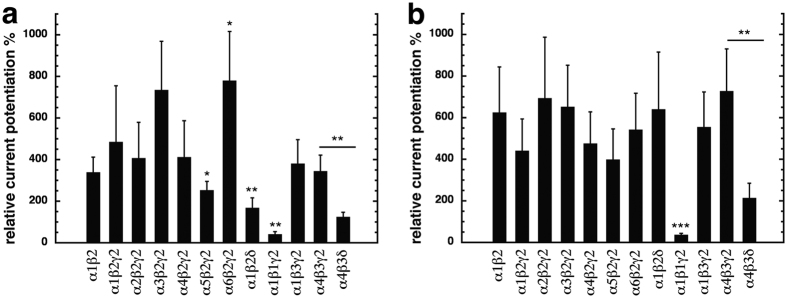
Subunit specificity of current potentiation in different GABA_A_ receptors. Different subunit combinations were expressed in *Xenopus* oocytes. Potentiation of GABA currents at a GABA concentration eliciting 0.5–1.5% of the maximal current amplitude was determined using 3 μM of compound 31 (**a**) or 132 (**b**). Bars indicate mean ± SD, n = 4–11.

**Figure 8:**
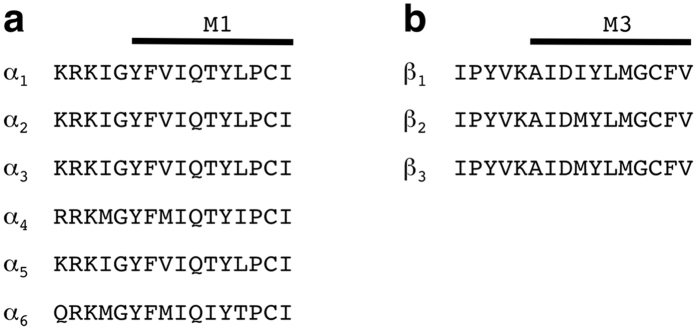
Alignment of the rat amino acid residue sequences of different α (a) and β (b) subunit isoforms. (**a**) Sequences preceding M1 and the first part of M1 in α subunits are shown. (**b**) Sequences preceding M3 and the first part of M3 in β subunits are shown.

From the above experiments we inferred that the β+/α− TMD subunit interfaces mediate potentiation of compounds 31 and 132. We also wanted to know if potentiation by these compounds depends on subunit isoforms. First we replaced the α_1_ subunit by different α subunit isoforms: α_1_β_2_γ_2_, α_2_β_2_γ_2_, α_3_β_2_γ_2_, α_4_β_2_γ_2_, α_5_β_2_γ_2_ and α_6_β_2_γ_2_ (Fig. 7). Compound 31 displayed a similar degree of potentiation in α_1_β_2_γ_2_ receptors 485 ± 230% (mean ± SD, n = 11), α_2_β_2_γ_2_ receptors, 407 ± 172% (mean ± SD, n = 4, p > 0.05, *Tukey posthoc test*), α_3_β_2_γ_2_ receptors, 735 ± 234%, (mean ± SD, n = 5, p > 0.05, *Tukey posthoc test*), and α_4_β_2_γ_2_ receptors, 412 ± 175% (mean ± SD, n = 4, p > 0.05, *Tukey posthoc test*). The α_5_β_2_γ_2_ receptor showed a significant decrease in potentiation compared to α_1_β_2_γ_2_ receptors to 253 ± 92% (mean ± SD, n = 8; p = 0.023, *Tukey posthoc test*), and the α_6_β_2_γ_2_ receptor an increase in potentiation, amounting to 780 ± 236% (mean ± SD, n = 4; p = 0.048, *Tukey posthoc test*). This discrepancy in modulation of α_5_β_2_γ_2_ and α_6_β_2_γ_2_ receptors maybe explained by the fact that 3 of the first 7 residues of M1 located at the minus side of the α subunit are different (Fig. 8a). Compound 132 produced similar potentiation in receptors with all α subunits tested. Amounting to 415 ± 126% (mean ± SD, n = 6) in α_1_β_2_γ_2_ receptors, 694 ± 293% (mean ± SD, n = 4, p > 0.05, *Tukey posthoc test*) in α_2_β_2_γ_2_ receptors, 652 ± 215% (mean ± SD, n = 4, p > 0.05, *Tukey posthoc test*) in α_3_β_2_γ_2_ receptors, 476 ± 152% (mean ± SD, n = 4, p > 0.05, *Tukey posthoc test*) in α_4_β_2_γ_2_ receptors, 399 ± 147% (mean ± SD, n = 4, p > 0.05, *Tukey posthoc test*) in α_5_β_2_γ_2_ receptors, and 542 ± 175% (mean ± SD, n = 4, p > 0.05, *Tukey posthoc test*) in α_2_β_2_γ_2_ receptor type. These results indicate that although the type of α subunit has differential effects on potentiation between compounds 31 and 132, these compounds modulate all receptor subtypes studied.

Next, we examined the role of the β subunit, replacing the β_2_ by β_1_ or β_3_. For compounds 31 and 132, α_1_β_3_γ_2_ receptors showed a similar potentiation as α_1_β_2_γ_2_ receptors. Amounting to 381 ± 115% for compound 31 and 555 ± 169% for compound 132 (mean ± SD, n = 4, p > 0.05, *Tukey posthoc test*). In the case of α_1_β_1_γ_2_ receptors, potentiation by both compounds was significantly reduced compared to that in α_1_β_2_γ_2_, 41 ± 13% for compound 31 and 37 ± 7% for compound 132 (n = 4; p = 0.0023, *Tukey posthoc test* for compound 31; n = 4; p = 0.0004, *Tukey posthoc test,* for compound 132). These results indicate that the type of β subunit is important for the potentiation by both compounds. It is interesting to note in this context that β_1_ and β_2_/β_3_ differ not only in the residue 265, but also in the fourth residue of M3 predicted to be close to the latter residue (Fig. 8b).

When the γ_2_ subunit was omitted, no statistical difference was observed for either compound tested. Compound 31 displayed a similar degree of potentiation between α_1_β_2_γ_2_ receptors and α_1_β_2_ receptors, 339 ± 73% (mean ± SD, n = 4, p > 0.05, *Tukey posthoc test*). Compound 132 also showed a similar potentiation between both receptors, potentiation in α_1_β_2_ receptors amounting to 625 ± 219% (mean ± SD, n = 4, p > 0.05, *Tukey posthoc test*). When the γ_2_ subunit was replaced by a δ subunit, in the case of α_1_β_2_δ receptors, potentiation was affected only in the case of compound 31, where a significant reduction was observed relative to α_1_β_2_γ_2_ receptors. Where potentiation amounted to 168 ± 48% (mean ± SD, n = 4, p = 0.0026, *Tukey posthoc test*) for compound 31, and 640 ± 275% (mean ± SD, n = 4, p > 0.05, *Tukey posthoc test*) for compound 132. In α_4_β_3_δ receptors, potentiation by both compounds was statistically reduced relative to α_4_β_3_γ_2_ receptors. For compound 31 potentiation amounted to 345 ± 77% (mean ± SD, n = 4) in α_4_β_3_γ_2_ receptors, and was reduced to 125 ± 22% (mean ± SD, n = 4, p = 0.0015, *Tukey posthoc test*) in α_4_β_3_δ receptors. Potentiation of compound 132 in α_4_β_3_γ_2_ receptors was 728 ± 203% (mean ± SD, n = 4), and was reduced to 214 ± 71% (mean ± SD, n = 4, p = 0.0031 *Tukey posthoc test*, for compound 132) in α_4_β_3_δ receptors.

Previous work^36^ showed that potentiation by 4-O-methylhonokiol was dependent on the α subunit in a similar fashion as compound 31, since modulation was reduced by receptors containing α_5_ and α_6_ subunits. The type of β subunit was also important, as the presence of the β_1_ subunit strongly reduced potentiation by this compound. On the contrary, the presence of a γ or a δ subunit did not affect potentiation.

### Anesthetic activity in tadpoles

**Figure 9:**
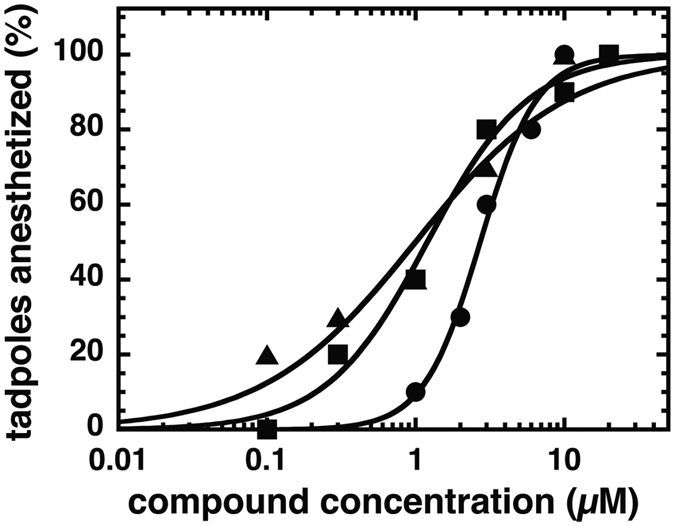
Concentration-response curves for loss of righting reflexes (LORR) in tadpoles for compounds 31 (closed circle), 132 (closed square), and 4-O-methylhonokiol (closed triangle). The percent of animals anesthetized is plotted against aqueous anesthetic concentration, overlaid with logistic fits. Each point represents data from ten animals. Data were fitted to a Hill equation.

The anesthetic activity for compounds 31, 132 and 4-O-methylhonokiol was determined as loss of righting reflex (LoRR) in *Xenopus* tadpoles, Fig. 9 shows the concentration dependence curve for each. Compound 31 yielded an EC_50_ of 2.7 μM (95% confidence interval = 2.0 to 3.7 μM), while the EC_50_ compound 132 was 1.2 μM (95% confidence interval = 0.73 to 2.0 μM), and 4-O-methylhonokiol EC_50_ = 1.0 μM (95% confidence interval = 0.46 to 2.2 μM). For comparison, the EC_50_ for propofol-induced LoRR in tadpoles is 1.3 μM^41^. Anesthesia was fully reversible for compound 31; for animals tested with compound 132, recovery was minimal at concentrations above 3 μM. For 4-O-methylhonokiol, animals tested at a concentration of 10 μM did not recover, whereas recovery was complete at 3 μM and lower concentrations.

## Discussion

Here we functionally characterized compounds 31, 132 and further investigated the properties of 4-O-methylhonokiol. All three compounds are potent allosteric potentiators of α_1_β_2_γ_2_ GABA_A_ receptors that do not act at site 1. We suspected that they instead act through the low affinity TMD site(s) for benzodiazepines (site 3). Indeed, potentiation by compounds 31, 132 and 4-O-methylhonokiol was abolished in the triple mutant receptor α_1_S269Iβ_2_N265Iγ_2_S280I as well as by the single mutation β_2_N265I, but unaffected by the homologous mutations α_1_S269I and γ_2_S280I. Assuming that these TM2 mutations alter drug actions through local steric effects in adjacent TMD interfacial sites, our results indicate that of the five such sites, only the two β+/α− interfaces 1 and 2 mediate the potentiating effects of these three compounds.

We further dissected the contribution of the individual β+/α- subunit interfaces using concatenated subunit assemblies. The γβ+/α−β interface (interface 1) and αβ+/α−γ interface (2) participated differently in modulation by the three compounds studied. For all compounds the contribution of the interface 1 to drug modulation is apparently greater than that of the interface 2.

We and others have shown that the intravenous anesthetics etomidate, propofol and pentobarbital also act via TMD interfacial sites^26^,^27^,^28^,^29^,^37^. In α_1_β_2_γ_2_ receptors, β_2_N265I reduced potentiation by all compounds, α_1_S269I reduced potentiation exclusively by pentobarbital, and the γ_2_S280I mutation increased potentiation by etomidate, while reducing potentiation by propofol and pentobarbital^37^. Different sets of residues located at subunit interfaces have been photo-labeled by etomidate, barbiturate, and propofol analogs, revealing that some anesthetics selectively bind within different TMD interfaces^23^,^24^,^33^,^42^,^43^. Additionally, mutations of residues at the β+/α− subunit interface affecting anesthetic action have been shown to affect modulation by valerenic acid. This suggests that the binding pocket for this compound is also at or near the anesthetics binding site^44^. Other subunit interfaces were not investigated.

Work by our group using receptor concatenation determined that both β+/α− subunit interfaces participated equally in modulation by propofol. In contrast, modulation by etomidate was found to be more affected by the γβ+/α−β interface site (interface 1) than the αβ+/α−γ site (interface 2)^37^. Interestingly, studies using another mutation (β_2_M286W) and different concatenated subunit assemblies suggest that etomidate interactions are equivalent in the two β+/α− sites of α_1_β_2_γ_2_ receptors^45^.

The homologs of β_2_N265 in β_1_ and β_3_ are serine and asparagine, respectively, and this single residue dramatically influences sensitivity to loreclezole^39^, etomidate and propofol^26^,^28^,^29^,^39^,^40^. Potentiation by compound 31 was strongly affected by this mutation while that by compound 132 was affected less. On the other hand, potentiation by 132 was severely reduced in α_1_β_1_γ_2_ receptors, compared to α_1_β_2_γ_2_ receptors. Potentiation by 4-O-methylhonokiol is also reduced by the β_2_N265S mutation or substitution of β_1_ for β_2_^36^.

In subunit specificity studies, compounds 31 and 132 potentiated receptors containing the α_4_ and α_6_ subunits, contrasting with benzodiazepine site 1 agonists. Both compounds also potentiated receptors carrying the δ subunit, although to different degrees. Thus, these compounds not only acted at receptors shown to be located synaptically as α_1_β_2–3_γ_2_, α_2_β_2_γ_2_ and α_3_β_2_γ_2_^46^, but also at α_5_β_2_γ_2_ receptors and receptors containing the δ subunit, which are all located extra-synaptically^5^,^46^,^47^. Similarly, SJM-3 modulates both synaptic and extrasynaptic receptors^35^.

The similarities between the three compounds we studied and the clinical anesthetics propofol, etomidate and pentobarbital suggested their possible use as sedative-hypnotics in animals. Indeed, all three compounds induced reversible loss of righting reflexes (LoRR) in *Xenopus laevis* tadpoles with EC_50_s comparable to the anesthetics propofol^41^, and etomidate^48^. However, LoRR was not reversible with high concentrations of compound 132 and 4-O-methylhonokiol.

In summary, the newly identified compounds 31 and 132 modulate both synaptic and extrasynaptic GABA_A_ receptors at molecular sites different from the classical benzodiazepine pocket. These compounds, together with 4-O-methylhonokiol, act through β+/α− TMD interfaces, with strongest effects through the interface 1. These compounds potently produced LoRR in aquatic animals and thus may be useful lead compounds in the search for novel anesthetic, sedative-hypnotic or anxiolytic drugs.

## Methods

### Construction of mutated receptor subunits

The point mutations α_1_S269I, β_2_N265I, β_2_N265S and γ_2_S280I were prepared using the QuikChange^TM^ mutagenesis kit (Stratagene, Agilent Technologies, Basel, Switzerland).

### Construction of concatenated subunits

Construction of tandem and triple subunit cDNAs. The tandem construct γ_2_-β_2_, and triple construct α_1_-β_2_-α_1_ has been described previously^12^. Site-directed mutagenesis of β_2_N265 to I was done in the tandem construct and the triple construct using the QuikChange^TM^ mutagenesis kit (Stratagene, Agilent Technologies, Basel, Switzerland).

### Expression of GABA_A_ receptors in Xenopus oocytes

Capped cRNAs were synthesized (Ambion, Austin, TX, USA) from the linearized plasmids with a cytomegalovirus promotor (pCMV vectors) containing the different subunits, respectively. A poly-A tail of about 400 residues was added to each transcript using yeast poly-A polymerase (United States Biologicals, Cleveland, OH, USA). The concentration of the cRNA was quantified on a formaldehyde gel using Radiant Red stain (Bio-Rad) for visualization of the RNA. Known concentrations of RNA ladder (Invitrogen) were loaded as standard on the same gel. cRNAs were precipitated in ethanol/isoamylalcohol 19:1, the dried pellet dissolved in water and stored at −80 °C. cRNA mixtures were prepared from these stock solutions and stored at −80 °C.

Animal experiments were carried out in strict accordance to the Swiss ethical guidelines, and have been approved by the local committee of the Canton Bern Kantonstierarzt, Kantonaler Veterinärdienst Bern (BE85/15). Surgery was done under anesthesia, and all efforts were made to diminish animal suffering. Xenopus laevis oocytes were prepared, injected and defolliculated as described previously^49^,^50^. Oocytes were injected with 50 nL of the cRNA solution containing wild type or mutated rat α_1_, β_2_ and γ_2_ subunits of the GABA_A_ receptors at a concentration of 10 nM:10 nM:50 nM^51^. For concatenated tandem and triple constructs, cRNA combinations ratios of 25: 25 nM were used. Injected oocytes were incubated in modified Barth’s solution at 18 °C for at least 24 h before the measurements.

### Functional characterization of GABA_A_ receptors

Currents were measured using a modified two-electrode voltage clamp amplifier Oocyte clamp OC-725 (Warner Instruments) in combination with a XY-recorder (90% response time 0.1 s) or digitized at 100 Hz using a PowerLab 2/20 (AD Instruments) using the computer programs Chart (ADInstruments GmbH, Spechbach, Germany). Tests with a model oocyte were performed to ensure linearity in the larger current range. The response was linear up to 15 μA. The holding potential was −80 mV. The perfusion medium contained 90 mM NaCl, 1 mM KCl, 1 mM MgCl_2_, 1 mM CaCl_2_, and 5 mM Na-HEPES (pH 7.4). Concentration response curves for the compounds were fitted with the equation I(c) = I_max_/[1 + (EC_50_/c)^n^], where c is the concentration of the compound, EC_50_ the concentration eliciting half-maximal current amplitude, I_max_ is the maximal current amplitude, I the current amplitude, and n is the Hill coefficient. Maximal current amplitudes (I_max_) were obtained from the fits of the concentration-response curves. For all receptors studied, modulation was measured at a GABA concentration eliciting 0.5–1.5% of the maximal GABA current amplitude. GABA was applied twice alone for 20–60 s, and then in combination with the different compounds for 45 s or 1 min. The duration of washout periods was 4 min in between agonist or agonist/drug aplications to prevent receptor desentization. At the beginning of the experiments, GABA applications were repeated when the elicited current amplitude altered by >5%. Potentiation was calculated by the following equation: (I_Modulator + GABA_/I_GABA_ − 1) * 100%. The perfusion solution was applied through a glass capillary with an inner diameter of 1.35 mm, the mouth of which was placed about 0.4 mm from the surface of the oocyte. This allowed fast changes in agonist concentration around the oocyte. The rate of change was estimated 70% in less than 0.5 s^14^. The perfusion system was cleaned between drug applications by washing with DMSO to avoid contamination. All media contained a final concentration of 0.5% DMSO (v/v) to ensure drug solubility.

All data are from at least two different batches of oocytes. Data represent mean ± SD. An unpaired *t test* was used to compare two means. One-way analysis of variance (ANOVA) was used for multiple comparisons followed by a *Tukey post hoc* test. *p < 0.05; **p < 0.01; ***p < 0.001.

### Loss of righting reflex assay in Xenopus tadpole

Animals were used and experiments were carried out with approval and according to the guidelines of the MGH Institutional Animal Care and Use Committee. General anesthetic potency was assessed in *Xenopus* laevis tadpoles as previously described^41^,^52^,^53^. In brief, groups of 10 tadpoles were placed in aqueous solutions containing compound 31, 132, or 4-O-methylhonokiol, and tested every five minutes for loss of righting reflexes (LoRR). Each animal was assigned a score of either awake or LoRR, and the percent of animals anesthetized was plotted against the concentration of the compound tested. Concentration-response data was fitted by non-linear least squares to logistic functions of the form Y = 1/(1 +10^((LogEC_50_ − Log[Drug])*HillSlope)) using Graphpad Prism 6. Results are reported as EC_50s_ and 95% confidence intervals.

## Additional Information

**How to cite this article**: Maldifassi, M. C. *et al*. Novel positive allosteric modulators of GABA_A_ receptors with anesthetic activity. *Sci. Rep.* **6**, 25943; doi: 10.1038/srep25943 (2016).

## References

[b1] Forman S. A. Molecular approaches to improving general anesthetics. Anesthesiol. Clin. 28, 761–771 (2010). 21074751 10.1016/j.anclin.2010.08.004 PMC2990980

[b2] Chitilian H. V., Eckenhoff R. G. & Raines D. E. Anesthetic drug development: novel drugs and new approaches. Surg. Neurol. Int. 4, S2–S10 (2013). 23653886 10.4103/2152-7806.109179 PMC3642742

[b3] Macdonald R. L. & Olsen R. W. GABA_A_ receptor channels. Annu. Rev. Neurosci. 17, 569–602 (1994). 7516126 10.1146/annurev.ne.17.030194.003033

[b4] Barnard E. A. . International union of pharmacology. XV. Subtypes of gamma-aminobutyric acid A receptors: classification on the basis of subunit structure and receptor function. Pharmacol. Rev. 50, 291–313 (1998). 9647870

[b5] Olsen R. W. & Sieghart W. International union of pharmacology. LXX. Subtypes of γ-aminobutyric acid type A receptors: classification on the basis of subunit composition, pharmacology, and function. Update. Pharmacol. Rev. 60, 243–260 (2008). 18790874 10.1124/pr.108.00505 PMC2847512

[b6] Sigel E. & Steinmann M. E. Structure, function, and modulation of GABA_A_ receptors. J. Biol. Chem. 287, 40224–40231 (2012). 23038269 10.1074/jbc.R112.386664 PMC3504738

[b7] Rabow L. E., Russek S. J. & Farb D. H. From ion currents to genomic analysis: recent advances in GABA_A_ receptor research. Synapse 21, 189–274 (1995). 8578436 10.1002/syn.890210302

[b8] Chang Y., Wang R., Barot S. & Weiss D. S. Stoichiometry of a recombinant GABA_A_ receptor. J. Neurosci. 16, 5415–5424 (1996). 8757254 10.1523/JNEUROSCI.16-17-05415.1996 PMC6578878

[b9] Farrar S. J., Whiting P. J., Bonnert T. P. & McKernan R. M. Stoichiometry of a ligand-gated ion channel determined by fluorescence energy transfer. J. Biol. Chem. 274, 10100–10104 (1999). 10187791 10.1074/jbc.274.15.10100

[b10] Tretter V., Ehya N., Fuchs & Sieghart K. W. Stoichiometry and assembly of a recombinant GABA_A_ receptor subtype. J. Neurosci. 17, 2728–2737 (1997). 9092594 10.1523/JNEUROSCI.17-08-02728.1997 PMC6573102

[b11] Baumann S. W., Baur R. & Sigel E. Subunit arrangement of gamma aminobutyric acid type A receptors. J. Biol. Chem. 276, 36275–36280 (2001). 11466317 10.1074/jbc.M105240200

[b12] Baumann S. W., Baur R. & Sigel E. Forced subunit assembly in α_1_β_2_γ_2_ GABA_A_ receptors. Insight into the absolute arrangement. J. Biol. Chem. 277, 46020–46025 (2002). 12324466 10.1074/jbc.M207663200

[b13] Baur R., Minier F. & Sigel E. A GABA_A_ receptor of defined subunit composition and positioning: concatenation of five subunits. FEBS Lett. 580, 1616–1620 (2006). 16494876 10.1016/j.febslet.2006.02.002

[b14] Sigel E., Baur R., Trube G., Mohler H. & Malherbe P. The effect of subunit combination of rat brain GABA_A_ receptors on channel function. Neuron. 5, 703–711 (1990). 1699569 10.1016/0896-6273(90)90224-4

[b15] Minier F. & Sigel E. Positioning of the α-subunit isoforms confers a functional signature to γ-aminobutyric acid type A receptors. Proc. Natl. Acad. Sci. USA 101, 7769–7774 (2004). 15136735 10.1073/pnas.0400220101 PMC419681

[b16] Sigel E. & Lüscher B. P. A closer look at the high affinity benzodiazepine binding site on GABA_A_ receptors. Curr. Top. Med. Chem. 11, 241–246 (2011). 21189125 10.2174/156802611794863562

[b17] Smith G. B. & Olsen R. W. Functional domains of GABA_A_ receptors. Trends Pharmacol. Sci. 16, 162–168 (1995). 7624971 10.1016/s0165-6147(00)89009-4

[b18] Baur R. . Covalent modification of GABA_A_ receptor isoforms by a diazepam analogue provides evidence for a novel benzodiazepine binding site that prevents modulation by these drugs. J. Neurochem. 106, 2353–2363 (2008). 18643789 10.1111/j.1471-4159.2008.05574.x

[b19] Ramerstorfer J. R. . The GABA_A_ receptor α+/β– interface: a novel target for subtype selective drugs. J. Neurosci. 31, 870–877 (2011). 21248110 10.1523/JNEUROSCI.5012-10.2011 PMC3182524

[b20] Walters R. J., Hadley S. H., Morris K. D. & Amin J. Benzodiazepines act on GABA_A_ receptors via two distinct and separable mechanisms. Nat. Neurosci. 12, 1274–1281 (2000). 11100148 10.1038/81800

[b21] Nishikawa K., Jenkins A., Paraskevakis I. & Harrison N. L. Volatile anesthetic actions on the GABA_A_ receptors: contrasting effects of α_1_(S270) and β_2_(N265) point mutations. Neuropharmacol. 42, 337–345 (2002). 10.1016/s0028-3908(01)00189-7 11897112

[b22] Chang C. S., Olcese R. & Olsen R. W. A single M1 residue in the β_2_ subunit alters channel gating of GABA_A_ receptor in anesthetic modulation and direct activation. J. Biol. Chem. 278, 42821–42828 (2003). 12939268 10.1074/jbc.M306978200

[b23] Li G. D. . Identification of a GABA_A_ receptor anesthetic binding site at subunit interfaces by photolabeling with an etomidate analog. J. Neurosci. 26, 11599–11605 (2006). 17093081 10.1523/JNEUROSCI.3467-06.2006 PMC6674783

[b24] Yip G. M. . A propofol binding site on mammalian GABA_A_ receptors identified by photolabeling. Nat. Chem. Biol. 9, 715–720 (2013). 24056400 10.1038/nchembio.1340 PMC3951778

[b25] Rudolph U. & Antkowiak B. Molecular and neuronal substrates for general anaesthetics. Nat. Rev. Neurosci. 5, 709–720 (2004). 15322529 10.1038/nrn1496

[b26] Belelli D., Lambert J. J., Peters J. A., Wafford K. & Whiting P. J. The interaction of the general anesthetic etomidate with the gamma-aminobutyric acid type A receptor is influenced by a single amino acid. Proc. Natl. Acad. Sci. USA 94, 11031–11036 (1997). 9380754 10.1073/pnas.94.20.11031 PMC23576

[b27] Krasowski M. D. . Propofol and other intravenous anesthetics have sites of action on the gamma-aminobutyric acid type A receptor distinct from that for isoflurane. Mol. Pharmacol. 53, 530–538 (1998). 9495821 10.1124/mol.53.3.530

[b28] Siegwart R., Krahenbuhl K., Lambert S. & Rudolph U. Mutational analysis of molecular requirements for the actions of general anaesthetics at the γ-aminobutyric acid type A receptor subtype, α_1_β_2_γ_2_. BMC Pharmacol. 3, 13 (2003). 14613517 10.1186/1471-2210-3-13 PMC280653

[b29] Stewart D. S., Pierce D. W., Hotta M., Stern A. T. & Forman S. A. Mutations at βN265 in γ-aminobutyric acid type A receptors alter both binding affinity and efficacy of potent anesthetics. PLoS One 9, e111470 (2014). 25347186 10.1371/journal.pone.0111470 PMC4210246

[b30] Jurd R. . General anesthetic actions *in vivo* strongly attenuated by a point mutation in the GABA(A) receptor β_3_ subunit. FASEB J. 2, 250–252 (2002). 12475885 10.1096/fj.02-0611fje

[b31] Hill-Venning C., Belelli D., Peters J. A. & Lambert J. J. Subunit-dependent interaction of the general anaesthetic etomidate with the gamma-aminobutyric acid type A receptor. Br. J. Pharmacol. 120, 749–756 (1997). 9138677 10.1038/sj.bjp.0700927 PMC1564523

[b32] Franks N. P. General anaesthesia: from molecular targets to neuronal pathways of sleep and arousal. Nat. Rev. Neurosci. 5, 370–386 (2008). 18425091 10.1038/nrn2372

[b33] Jayakar S. S. . Multiple propofol-binding sites in a γ-aminobutyric acid type A receptor (GABA_A_R) identified using a photoreactive propofol analog. J. Biol. Chem. 289, 27456–27468 (2014). 25086038 10.1074/jbc.M114.581728 PMC4183786

[b34] Middendorp S. J., Puthenkalam R., Baur R., Ernst M. & Sigel E. Accelerated discovery of novel benzodiazepine ligands by experiment-guided virtual screening. ACS Chem. Biol. 9, 1854–1859 (2014). 24960548 10.1021/cb5001873

[b35] Middendorp S. J., Maldifassi M. C., Baur R. & Sigel E. Positive modulation of synaptic and extrasynaptic GABA_A_ receptors by an antagonist of the high affinity benzodiazepine binding site. Neuropharmacol. 95, 459–467 (2015). 10.1016/j.neuropharm.2015.04.027 25963418

[b36] Baur R., Schuehly W. & Sigel E. Moderate concentrations of 4-O-methylhonokiol potentiate GABA_A_ receptor currents stronger than honokiol. Biochim. Biophys. Acta. 1840, 3017–3021 (2014). 24973566 10.1016/j.bbagen.2014.06.016

[b37] Maldifassi M. C., Baur R. & Sigel E. Functional sites involved in modulation of the GABA_A_ receptor channel by the intravenous anesthetics propofol, etomidate and pentobarbital. Neuropharmacol. 105, 207–214 (2016). 10.1016/j.neuropharm.2016.01.003 26767954

[b38] Miller P. S. & Aricescu A. R. Crystal structure of a human GABA_A_ receptor. Nature 512, 270–275 (2014). 24909990 10.1038/nature13293 PMC4167603

[b39] Wingrove P. B., Wafford K. A., Bain C. & Whiting P. J. The modulatory action of loreclezole at the γ-aminobutyric acid type A receptors is determined by a single amino acid in the β_2_ and β_3_ subunit. Proc. Natl. Acad. Sci. USA 91, 4569–4573 (1994). 8183949 10.1073/pnas.91.10.4569 PMC43827

[b40] Fernandez S. P. . Flavan-3-ol esters: new agents for exploring modulatory sites on GABA_A_ receptors. Br. J. Pharmacol. 165, 965–977 (2012). 21806603 10.1111/j.1476-5381.2011.01615.x PMC3312492

[b41] Stein M. . Azo-propofols: photochromic potentiators of GABA_A_ receptors. Angew. Chem. Int. Ed. Engl. 51, 10500–10504 (2012). 22968919 10.1002/anie.201205475 PMC3606271

[b42] Chiara D. C. . Mapping general anesthetic binding site(s) in human α_1_β_3_ γ-aminobutyric acid type A receptors with [^3^H]TDBzl-etomidate, a photoreactive etomidate analogue. Biochemistry 51, 836–847 (2012). 22243422 10.1021/bi201772m PMC3274767

[b43] Chiara D. C. . Specificity of intersubunit general anesthetic binding sites in the transmembrane domain of the human α_1_β_3_γ_2_ GABA_A_ receptor. J. Biol. Chem. 288, 19343–19357 (2013). 23677991 10.1074/jbc.M113.479725 PMC3707639

[b44] Luger D. . Identification of the putative binding pocket of valerenic acid on GABA_A_ receptors using docking studies and site-directed mutagenesis. Br. J. Pharmacol. 22, 5403–5413 (2015). 26375408 10.1111/bph.13329 PMC4988470

[b45] Guitchounts G., Stewart D. S. & Forman S. A. The two etomidate sites in α_1_β_2_γ_2_ gamma-aminobutyric acid type A receptors contribute equally and noncooperatively to modulation of channel gating. Anesthesiology. 116, 1235–1244 (2012). 22531336 10.1097/ALN.0b013e3182567df3 PMC3366439

[b46] Jacob T. C., Moss S. J. & Jurd R. GABA_A_ receptor trafficking and its role in the dynamic modulation of neuronal inhibition. Nat. Rev. Neurosci. 9, 331–343 (2008). 18382465 10.1038/nrn2370 PMC2709246

[b47] Farrant M. & Nusser Z. Variations on an inhibitory theme: Phasic and tonic activation of GABA_A_ receptors. Nature Rev. Neurosci. 6, 215–229 (2005). 15738957 10.1038/nrn1625

[b48] Husain S. S. . 2-(3-Methyl-3H-diaziren-3-yl)ethyl 1-(1-phenylethyl)-1H-imidazole-5-carboxylate: a derivative of the stereoselective general anesthetic etomidate for photolabeling ligand-gated ion channels. J. Med. Chem. 46, 1257–1265 (2003). 12646036 10.1021/jm020465v

[b49] Sigel E. Properties of single sodium channels translated by Xenopus oocytes after injection with messenger ribonucleic acid. J. Physiol. Lond. 386, 73–90 (1987). 2445971 10.1113/jphysiol.1987.sp016523 PMC1192451

[b50] Sigel E. & Minier F. The Xenopus oocyte: system for the study of functional expression and modulation of proteins. Mol. Nutr. Food Res. 49, 228–234 (2005). 15704243 10.1002/mnfr.200400104

[b51] Boileau A. J., Baur R., Sharkey L. M., Sigel E. & Czajkowski C. The relative amount of cRNA coding for γ_2_ subunits affects stimulation by benzodiazepines in GABA_A_ receptors expressed in Xenopus oocytes. Neuropharmacol. 43, 695–700 (2002). 10.1016/s0028-3908(02)00036-9 12367615

[b52] Desai R. . Contrasting actions of a convulsant barbiturate and its anticonvulsant enantiomer on the α_1_β_3_γ_2_ GABA_A_ receptor account for their *in vivo* effects. J. Physiol. 593, 4943–4961 (2015). 26378885 10.1113/JP270971 PMC4650410

[b53] Ge R. L. . Pharmacological studies of methoxycarbonyl etomidate’s carboxylic acid metabolite. Anesth. Analg. 115, 305–308 (2012). 22052979 10.1213/ANE.0b013e318239c6ca PMC3291806

[b54] Baumann S. W., Baur R. & Sigel E. Individual properties of the two functional agonist sites in GABA_A_ receptors. J. Neurosci. 23, 11158–11166 (2003). 14657175 10.1523/JNEUROSCI.23-35-11158.2003 PMC6741049

